# Effect of Inhaled β_2_-Agonist on Exhaled Nitric Oxide in Chronic Obstructive Pulmonary Disease

**DOI:** 10.1371/journal.pone.0157019

**Published:** 2016-06-03

**Authors:** Mostafa Amer, Jan Cowan, Andrew Gray, Ben Brockway, Jack Dummer

**Affiliations:** 1 Otago Respiratory Research Unit, Department of Medicine, University of Otago, Dunedin, New Zealand; 2 Department of Preventive and Social Medicine, University of Otago, Dunedin, New Zealand; University of Athens, GREECE

## Abstract

The fractional exhaled nitric oxide measured at an expiratory flow of 50mL/s (FE_NO_50) is a marker of airway inflammation, and high levels are associated with greater response to steroid treatment. In asthma, FE_NO_50 increases with bronchodilation and decreases with bronchoconstriction, the latter potentially causing an underestimate of the degree of airway inflammation when asthma worsens. It is unknown whether the same effect occurs in chronic obstructive lung disease (COPD). Likewise, it is not known whether changes in airway calibre in COPD patients alter flow-independent parameters describing pulmonary nitric oxide exchange, such as the maximal flux of nitric oxide (NO) from the proximal airway compartment (J’aw_NO_) and the distal airway/alveolar concentration of NO (CA_NO_). We recruited 24 patients with COPD and performed FE_NO_ analysis at multiple expiratory flows before and after treatment with inhaled β_2_-agonist bronchodilator therapy. For the 21 patients analysed, FE_NO_50 rose from 17.1 (1.4) ppb (geometric mean (geometric SD)) at baseline, to 19.3 (1.3) ppb after bronchodilator therapy, an increase of 2.2 ppb (95% CI, 0.7–3.6; *P* = 0.005). There were non-significant changes in flow-independent NO parameters. The change in FE_NO_50 correlated positively with the change in J’aw_NO_ (*r*_*s*_ = 0.67, *P* < 0.001; *r*_*s*_ = 0.62, *P* = 0.002 before and after correction for axial back-diffusion respectively) following bronchodilation. Inhaled bronchodilator therapy can increase exhaled nitric oxide measurements in COPD. The standardisation of inhaled bronchodilator therapy before FE_NO_ analysis in COPD patients should therefore be considered in both research and clinical settings.

## Introduction

The fraction of exhaled nitric oxide (FE_NO_50) is a non-invasive biomarker of inflammation associated with T-helper type 2 cells and eosinophils in the airways, which typically occurs in asthma and responds to inhaled corticosteroid (ICS) [1–3]. FE_NO_50 is therefore useful for predicting whether or not a patient with airways disease will respond to ICS, with higher levels being associated with greater responsiveness [4].

Nonetheless, as a clinical tool, FE_NO_50 has some limitations, and one of these is that it is influenced by airway calibre. Previous studies in asthma patients have shown that administration of inhaled salbutamol causes an increase in FE_NO_50 of approximately 10% [5]. More recent studies have shown that acute bronchoconstriction is associated with a drop in FE_NO_50, a reduction in FEV_1_ of around 30% being associated with a similar reduction in FE_NO_50 [6, 7]. This is problematic because, at a time of deteriorating asthma control, bronchoconstriction may result in a falsely reassuring FE_NO_50 implying minimal airway inflammation when, in fact, the inflammatory state has worsened.

Many patients with COPD exhibit bronchodilator reversibility [8] but little is known about the effect of changes in airway calibre on FE_NO_50 in COPD. There is some evidence to suggest that a greater degree of reversibility of airway obstruction is associated with an elevated FE_NO_50 and eosinophilic airway inflammation [9, 10]. COPD patients with this phenotype of high FE_NO_50 and airway eosinophilia are also more likely to respond to corticosteroid [11, 12]. At present, the effect of change in airway calibre on FE_NO_50 in COPD patients is uncertain. This is important to investigate because it may be more difficult to detect corticosteroid-responsive COPD patients if FE_NO_50 measurements are performed when their airways are constricted, and FE_NO_50 is lower than it otherwise might be.

COPD is typically associated with inflammation of the distal airways, so measures of nitric oxide concentration or production in the distal airways may be of value in COPD [13]. Using a two-compartment model of pulmonary nitric oxide (NO) exchange, flow-independent NO parameters can be derived from the measurement of exhaled nitric oxide concentration at multiple expiratory flows: the maximal flux of nitric oxide (NO) from the proximal airway compartment (J’aw_NO_) and the distal airway/alveolar concentration of NO (CA_NO_) [14]. More recently, a simplified method has been proposed, using only two expiratory flows, to determine surrogate markers of J’aw_NO_ and CA_NO_: the area under the curve of the NO concentration versus time plot (AUC-NO) at the expiratory flow of 200 mL/s (AUC_200_) represents the CA_NO_, and the difference in AUC-NO between the 50 and 200 mL/s exhalations (ΔAUC_50-200_) represents the J’aw_NO_ [15]. The effects of bronchodilation on these flow-independent NO exchange parameters, in patients with COPD, is unknown.

We hypothesised that, in COPD patients, FE_NO_50 would increase after administration of inhaled β_2_-agonist. The primary aim of the study was to determine any change in FE_NO_50 following the administration of bronchodilator. The secondary aim was to determine any change in flow-independent NO parameters following the administration of bronchodilator.

## Methods

### Participants

Twenty-four patients with COPD were recruited and attended a single visit between December 2014 and January 2015 inclusive. Patients were aged 45 years or older, had a smoking history of more than 10 pack years, a post-bronchodilator FEV_1_/FVC of less than 70% and FEV_1_ < 80% predicted. Patients had stable COPD with no exacerbations or use of antibiotics in the two weeks preceding study participation. For each patient, a history of current and past respiratory symptoms, smoking history and medications were obtained, and beclomethasone dipropionate (BDP) equivalents were calculated as described previously [16]. A modified Medical Research Council (mMRC) dyspnoea score [17] and COPD assessment test (CAT) [18] were also completed. Patients with diagnosed lung cancer, bronchiectasis, or other significant co-morbidity were excluded from the study, as were patients unable to perform the 50mL/s exhaled nitric oxide (FE_NO_50) manoeuvres in accordance with American Thoracic Society (ATS) guidelines [19]. A study protocol is provided for further information (S1 Protocol). This study was registered at the Australian New Zealand Clinical Trials Registry: ACTRN12616000140459, it was approved by the Northern B Health and Disability Ethics Committee (reference 14/NTB/164), and all patients gave written, informed consent. Māori consultation was undertaken with Ngāi Tahu.

### Procedures

Participants performed the following sequence of tests to allow for the known effects of spirometry on exhaled nitric oxide measurements: (1) FE_NO_50 analysis; (2) FE_NO_ analysis at multiple expiratory flows; and (3) spirometry. Forty-five minutes after spirometry, 400mcg salbutamol was administered via spacer, and, 15 minutes later, steps 1–3 were repeated. This sequence of tests allowed one hour to pass between baseline spirometry and post-bronchodilator FE_NO_ analysis, so the former did not affect the latter [5, 20, 21]. Fifteen minutes elapsed between administration of salbutamol and the second set of tests to allow bronchodilation to occur fully [22, 23]. All subjects were required to withhold tiotropium for 24 hours and all other inhalers for 12 hours prior to attendance, and current smokers were required to abstain from smoking within one hour of the study visit as currently recommended [19].

A chemiluminescence nitric oxide analyser (NOA 280i; Sievers, Boulder, CO) was used to measure FE_NO_50 as per ATS guidelines [19], and FE_NO_ was measured at 100, 150, 200, and 250 mL/s as described previously [14, 15]. Briefly, patients performed two exhalations at each expiratory flow, by inhaling NO-free air and exhaling against resistance to increase mouth pressure to 10cmH_2_O, thereby closing the soft palate and isolating the nasopharynx [24]. Pressure and NO concentration were recorded simultaneously for each exhalation manoeuvre, and subjects were encouraged to maintain the required pressure and flow through a visual biofeedback system. The nitric oxide analyser was calibrated weekly with known NO concentration (50 parts per million) and zero NO gases, as per the manufacturer’s guidelines. To examine consistency in the measurement of FE_NO_ between subjects, a random sample of 40 NO plateaus at different expiratory flows were subsequently re-read by a trained investigator blinded to the subject and test phase (pre- or post-bronchodilator).

CA_NO_ and J’aw_NO_ were calculated using the slope-intercept method with and without adjustment for axial back-diffusion [14]. AUC_200_ and ΔAUC_50-200_ were also calculated according to the recently published method [15].

Spirometry (FEV_1_) was performed using a flow-volume device (VMax 1022; SensorMedics; Yorba Linda, CA) according to current standards [22], and used to determine reversibility [21] and the degree of airflow limitation [25]. The spirometer was calibrated daily with a 3L calibration syringe prior to subject testing.

### Statistical analysis

Summary statistics are provided for all patient characteristics of interest and all clinical measures. Where continuous variables are log-normally distributed, geometric means and geometric standard deviations are provided. The changes in pre- and post-bronchodilator measurements were reviewed to ascertain their distributions, and, given no indication that changes were non-normally distributed, compared with paired t-tests using untransformed data. The associations between the change in FE_NO_50 level and change in FEV_1_ and between other pairs of continuous variables were assessed by Spearman’s rank correlation as it was not felt that linearity could be assumed a priori. Two-sided p values <0.05 were considered statistically significant. Statistical analyses were performed using GraphPad Prism version 6.05 for Windows (GraphPad Software, La Jolla California USA).

## Results

**Fig 1:**
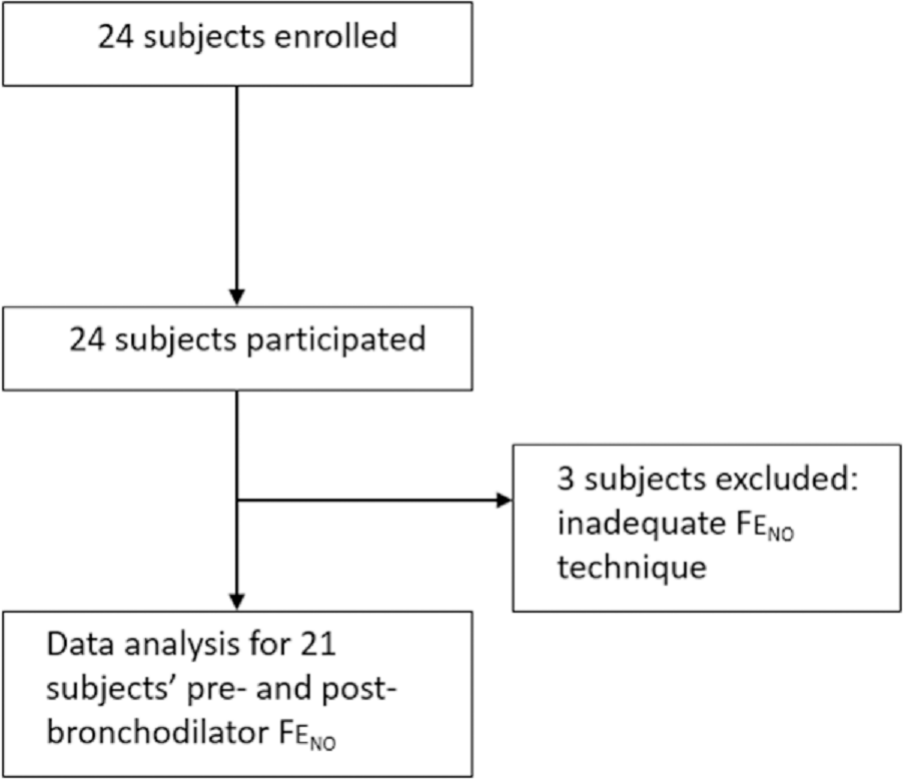
Study profile.

Twenty-four participants were recruited to the study, of whom three were excluded from the analysis because their FE_NO_50 exhalations did not meet ATS criteria for acceptable manoeuvres (Fig 1). The characteristics of the remaining 21 subjects are shown below (Table 1), and a minimal dataset is available as supporting information (S1 Table).

**Table 1:** Subject characteristics.

n (female/male)	21 (10/11)
Age, years	68 ± 10
NZ European, n (%)	20 (95)
Māori, n (%)	1 (5)
Body mass index, kg/m^2^	25.4 ± 4.3
Current smoker, n (%)	5 (24)
Ex-smoker, n (%)	16 (76)
Smoking pack-years	33 ± 14
Inhaled steroid, n (%)	17 (81)
BDP equivalent	1062 ± 847
mMRC score	1.8 ± 1.0
CAT score	19 ± 6
Post-bronchodilator FEV_1_/FVC ratio (%)	41.8 ± 11.2
Post-bronchodilator FEV_1_ (L)	1.15 ± 0.33
Post-bronchodilator FEV_1_ (% predicted)	43.3 ± 12.5
Reversible airflow obstruction,^‡^ n (%)	5 (24)
GOLD group A,^ψ^ n (%)	3 (14)
GOLD group B,^ψ^ n (%)	1 (5)
GOLD group C,^ψ^ n (%)	6 (29)
GOLD group D,^ψ^ n (%)	11 (52)

Data are presented as mean ± SD unless stated otherwise.

^‡^ Based on ≥ 12% and 200mL increase in FEV_1_ post-bronchodilator compared to baseline [21].

^ψ^ Global initiative for chronic Obstructive Lung Disease (GOLD) classification [25].

**Fig 2:**
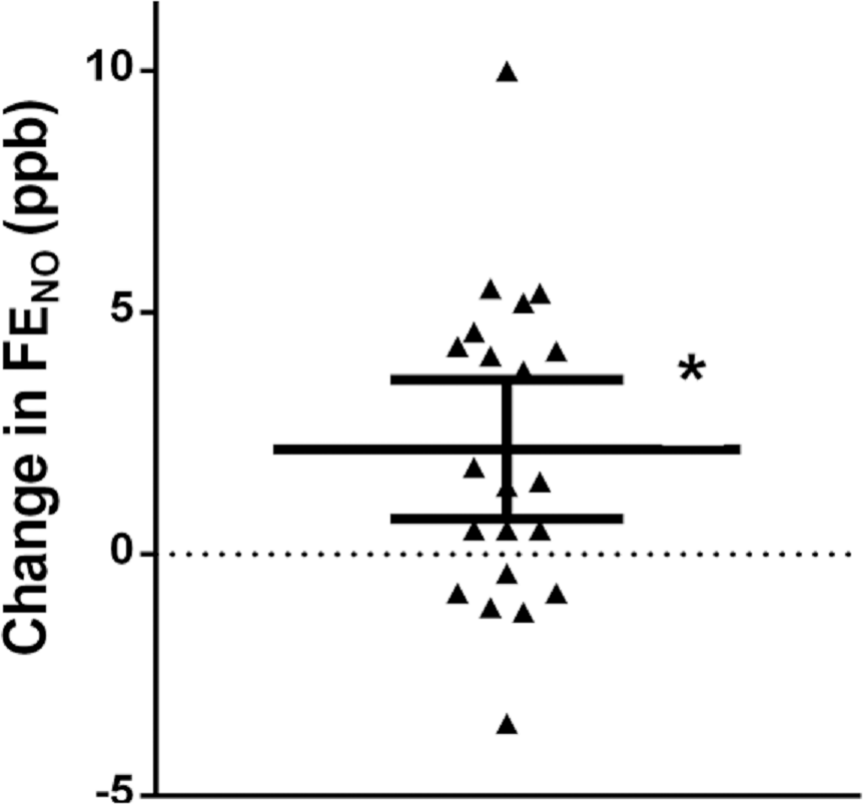
Plot showing the individual values and the mean with 95% confidence interval post-bronchodilator changes in FE_NO_50, ppb. (*P = 0.005 for the change in FE_NO_50).

FE_NO_50 rose from 17.1 (1.4) ppb (geometric mean (geometric SD)) at baseline, to 19.3 (1.3) ppb after bronchodilator therapy, an increase of 2.2 ppb (95% CI, 0.7–3.6; *P* = 0.005) (Fig 2). There were non-significant changes in the flow-independent NO parameters (Table 2).

**Table 2:** Pre- and post-bronchodilator spirometry, FE_NO_50 and flow-independent pulmonary NO parameters in 21 patients with COPD.

	Pre-bronchodilator	Post-bronchodilator	P-value^‡^
FEV_1_ (L)	1.00 ± 0.26	1.15 ± 0.33	**< 0.001**
FVC (L)	2.50 ± 0.64	2.81 ± 0.76	**< 0.001**
FE_NO_50 (ppb)^§^	17.1 ± 1.4	19.3 ± 1.3	**0.005**
J’aw_NO_ (pL/s)^Ҩ^	872 ± 866	924 ± 760	0.54
C_ANO_ (ppb) ^Ҩ^	3.2 ± 2.0	2.9 ± 2.2	0.44
J’aw_NO_ (pL/s)^Ψ^	1316 ± 1111	1489 ± 1295	0.09
C_ANO_ (ppb)^Ψ^	2.2 ± 1.6	1.7 ± 1.4	0.34
ΔAUC_50-200_ (ppb/s)	13.0 ± 8.7	15.6 ± 11.8	0.11
AUC_200_ (ppb/s)	7.6 ± 5.7	7.7 ± 6.2	0.85

Data are presented as mean ± SD unless stated otherwise.

^‡^Pre- vs post-bronchodilator

^§^ geometric mean ± geometric SD.

^Ҩ^Calculated using the slope-intercept method of Tsoukias and George [26].

^Ψ^Adjusted for axial back-diffusion using the Condorelli equation [14].

AUC: area under the curve of the NO concentration vs time plot–between the 50 and 200 mL/s exhalations (ΔAUC_50-200_), and at the 200 mL/s exhalation (AUC_200_) [15].

**Fig 3:**
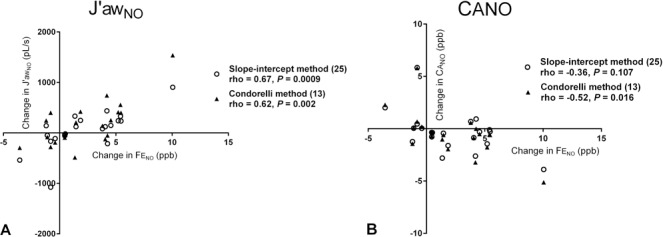
Scatter plots showing the correlation between the individual post-bronchodilator changes in FE_NO_50, and J’aw_NO_, pL/s (A), and CA_NO_, ppb (B), relative to baseline.

Both with and without adjustment for axial back-diffusion, the change in FE_NO_50 correlated positively with the change in J’aw_NO_ (*r*_*s*_ = 0.62, *P* = 0.002; *r*_*s*_ = 0.67, *P* < 0.001 respectively) and negatively with the change in CA_NO_ (*r*_*s*_ = 0.52, *P* = 0.02; *r*_*s*_ = 0.36, *P* = 0.11) following adminstration of inhaled β_2_-agonist (Fig 3). We did not observe a similar relationship between the change in FE_NO_50 and change in FEV_1_ (*r*_*s*_ = 0.19, *P* = 0.42).

The ΔAUC_50-200_ correlated with the J’aw_NO_ calculated using the slope-intercept method (*r*_*s*_ = 0.74, *P* < 0.001; *r*_*s*_ = 0.72, *P* < 0.001 pre- and post-bronchodilator respectively) [26]. The ΔAUC_50-200_ also correlated with the J’aw_NO_ when calculated using the Condorelli adjustment (*r*_*s*_ = 0.82, *P* < 0.001; *r*_*s*_ = 0.79, *P* < 0.001 pre- and post-bronchodilator respectively) [14]. There was a correlation between the AUC_200_ and the CA_NO_ calculated using the slope-intercept method [26] (*r*_*s*_ = 0.59, *P* = 0.005; *r*_*s*_ = 0.58, *P* = 0.006 pre- and post-bronchodilator respectively). No correlation was observed between the AUC_200_ and the CA_NO_ calculated using the Condorelli adjustment (*r*_*s*_ = 0.23, *P* = 0.32; *r*_*s*_ = 0.26, *P* = 0.26 pre- and post-bronchodilator respectively) [14].

## Discussion

FE_NO_50 increased by 2.2 ppb, or 13% of baseline levels, in COPD patients after administration of inhaled β_2_-agonist (*P* = 0.005). This finding is consistent with a number of previous studies of asthma patients. Silkoff *et al* previously showed that, after administration of inhaled β_2_-agonist, FE_NO_50 increased by approximately 10% in patients with asthma [5]. More recent studies of asthmatic patients showed that an acute reduction in airway calibre led to a parallel drop in FE_NO_50 [6, 7]. Other studies, however, have shown no change in FE_NO_ with changes in airway calibre in asthma and COPD [27, 28]. Any increase in FE_NO_50 after inhaled β_2_-agonist is most likely explained by the changes in airway wall dynamics that accompany the changes in airway calibre and influence NO diffusion. Since J’aw_NO_ is proportional to airway NO diffusion capacity (Daw_NO_), an increase in airway surface area and reduction in wall thickness through bronchodilation would be expected to increase Daw_NO_, and in turn, J’aw_NO_ and FE_NO_50 [6].

While the above mechanism is plausible, it should be noted that, in COPD, the distribution of nitric oxide production in the airways is incompletely understood, and may be quite different from that observed in asthma [29]. The increase in FE_NO_50 in response to inhaled β_2_-agonist may therefore be a result of different mechanisms in asthma and COPD. Proposed alternative mechanisms include the increased recruitment of airways, indicated by the increase in FVC, which may lead to increased release of “trapped” nitric oxide from recently constricted and hypoventilated airways (4, 5). There is also some *in vitro* evidence that salbutamol directly upregulates inducible nitric oxide synthase in bronchial epithelial cells and, if this occurs *in vivo*, it could result in increased airways production of nitric oxide and a rise in FE_NO_50 [30].

We were unable to show a relationship between change in FEV_1_ and change in FE_NO_50. This was in contrast to previous work demonstrating a positive correlation between changes in these parameters after bronchoconstriction in asthma [31]. Our study participants had a mean post-bronchodilator FEV_1_ of only 1.15 litres, so absolute changes in FEV_1_ before and after salbutamol were small and close to the accuracy limits of spirometry. This may have made it more difficult to determine any relationship between change in FEV_1_ and change in FE_NO_50.

CA_NO_ is commonly partitioned from J’aw_NO_ by a two-compartment model, requiring the subject to exhale at three different flows [14, 26]. In contrast, the AUC-NO has recently been proposed as a simpler procedure for the patient in order to obtain surrogates of J’aw_NO_ and CA_NO_ by using only two expiratory flows [15]. We found a strong correlation between the ΔAUC_50-200_ and J’aw_NO_ calculated with and without the Condorelli adjustment. However, the correlation between the AUC_200_ and the CA_NO_ was weaker and was not observed when the Condorelli adjustment was applied. It has recently been noted, on modelling, that the conducting airways can make a significant contribution to the AUC_200_ and, because of this, it cannot be used to reliably estimate CA_NO_ [32]. Our experimental results are consistent with this.

We have found that, in patients with COPD, administration of a bronchodilator can significantly change FE_NO_50, an increase in FEV_1_ of over one-eighth of baseline resulting in a similar increase in FE_NO_50. Thus, FE_NO_50 may be underestimated in a patient if measurement is performed in the presence of bronchoconstriction. The absolute change in FE_NO_50 was too small, for the most part, to mask an individual’s change in inflammatory status using population-derived cut-points for the presence or absence of steroid-responsive eosinophilic airway inflammation at >50ppb or <25ppb respectively.

Despite having FE_NO_50 levels that are similar to those reported in subjects without lung disease [29], as many as two thirds of COPD patients have evidence of eosinophilic airway inflammation [33]. When assessing the effect of an intervention in such a group, a change of at least 20% has been recommended as indicating a significant rise or fall in FE_NO_50 [34]. In this context, it would be important to consider FE_NO_50 measurement in the presence of a standardised amount of inhaled bronchodilator treatment in order to avoid variability in the measurement of up to the 13% observed in this study.

Our findings support the recent suggestion, by Haccuria *et al*., that change in airway calibre should be listed amongst the patient factors that influence measurement of FE_NO_50 in future ATS guidelines [7]. The same authors also suggest that, in asthma, a biomarker of airway inflammation incorporating both FE_NO_50 and FEV_1_ may have potential in guiding ICS treatment where, as yet, the use of FE_NO_50 alone has been disappointing [35]. A similar case could be made for such a biomarker in COPD.

In conclusion, our study shows that administration of inhaled β-agonist increases FE_NO_50 in COPD patients. Therefore, when performing FE_NO_50 analysis in both research and clinical settings, in patients with COPD, the standardisation of pre-test bronchodilator therapy should be considered.

## Supporting Information

S1 Protocol Study protocol. (PDF)

S1 Table Minimal data set. (PDF)
