# Supplementary material for: Effect of Inhaled β2-Agonist on Exhaled Nitric Oxide in Chronic Obstructive Pulmonary Disease
Source: PLoS One. 2016 Jun 3;11(6):e0157019. doi: 10.1371/journal.pone.0157019 (PMC4892672; doi:10.1371/journal.pone.0157019)
Supplement: S1 Protocol — (PDF) [file pone.0157019.s001.pdf]

Effect of Bronchodilator Therapy on Exhaled Nitric Oxide Measurement in Chronic Obstructive Pulmonary Disease

-Protocol-

Background and Rationale

Chronic obstructive pulmonary disease (COPD) is a common condition and major health burden in New Zealand, resulting in more than 200,000 GP visits, and over $100 million in direct healthcare costs per annum [1]. It is an airways disease characterised by persistent – and usually progressive – airflow obstruction, which may be partially reversible with bronchodilators, and typically manifests as chronic cough, sputum production and increasing breathlessness that may lead to respiratory failure. The principles of stable COPD management comprise risk factor modification – such as cessation of cigarette smoking – and pharmacological treatment, namely bronchodilator and inhaled corticosteroid (ICS) therapy [2].

The use of ICS in COPD is controversial [3, 4], but this therapy is recommended for patients with severe airflow limitation [2] and is prescribed to around 70% of COPD patients, despite evidence that it is of minimal or no benefit [3]. ICS therapy does not significantly affect the rate of lung function deterioration or mortality compared to placebo [5, 6] or a long-acting β2-agonist [7], and the risk of moderate-severe exacerbations does not increase following stepwise withdrawal [4]. In addition, it has several drawbacks, including hoarseness and oral candidiasis – to which COPD patients are particularly susceptible [6] – and increased risk of pneumonia [8, 9]. Further, the inhalers required to administer ICS are expensive, with the price of an inhaler, typically providing one month of treatment, ranging from $15 to $109 [10]. Thus, it would be advantageous to target ICS therapy to the minority of COPD patients who show objective benefit [11, 12] – as this has the potential to improve patient outcomes, reduce exposure to treatment side-effects and reduce costs – and exhaled nitric oxide (FE_{NO}) measurement is a clinical test that may be helpful in this regard [13].

FE_{NO} – a non-invasive and relatively inexpensive breath test – is a marker of eosinophilic airway inflammation, which typically occurs in asthma [14], and it is this type of inflammation that is responsive to ICS. FE_{NO} is therefore useful for predicting whether or not a

patient with airways disease will respond to ICS, with high levels being associated with greater responsiveness to ICS [15].

Nonetheless, as a clinical tool, FE_{NO} has limitations. One of the reasons for this is that FE_{NO} levels appear to be influenced by airway calibre. A study in asthma patients showed that an acute reduction in airway diameter was associated with a drop in FE_{NO} levels [16]. This is problematic because, at a time of deteriorating asthma control, bronchoconstriction may act to reduce FE_{NO} levels thereby implying minimal airway inflammation, whereas the opposite may be true.

However, little is known about the effect of changes in airway calibre in COPD. There is some evidence to suggest that FE_{NO} levels are reduced in COPD patients with more severe airflow obstruction [17], while other studies have shown that elevated FE_{NO} levels are associated with eosinophilic airway inflammation, with increased sputum eosinophils and a greater degree of reversibility of airway obstruction [18]. Patients with this eosinophilic phenotype are also more likely to respond to steroid [13, 19]. However, to date, there are no studies examining the effect of changes in airway calibre on FE_{NO} levels in individual COPD patients. This is important to investigate because COPD patients with this phenotype are more likely to respond to ICS, but may be difficult to detect if FE_{NO} levels are measured when their airways are constricted, and FE_{NO} is lower than it otherwise might be.

Hypotheses

In patients with COPD:

- FE_{NO} levels increase following administration of bronchodilator
- There is an association between the change in FE_{NO} level and the change in forced expiratory volume in 1 second (FEV_1) following administration of bronchodilator

Aims

- Determine any change in FE_{NO} level following administration of bronchodilator in patients with COPD
- Determine the relationship between the change in FE_{NO} level and FEV_1 in these patients

Research Design and Methods

In this study, twenty patients with COPD will complete a single visit at the Otago Respiratory Research Unit. Patients will abstain from bronchodilators for 12-24 hours prior to attendance.

During the visit we will:

1. Provide study information and obtain patient consent
2. Take a focussed history, and record pulse and BP
3. Complete a modified Medical Research Council (mMRC) dyspnoea score [20] and COPD assessment test (CAT) [21]

	Visit Schedule	est. time
1)	Information and consent	15 min
2)	Demographics, history, pulse and BP	15 min
3)	FE_{NO} and MEF FE_{NO}	30 min
4)	Spirometry (FEV_1)	10 min
5)	Washout following spirometry – complete mMRC & CAT scores<br>**give bronchodilator at 45 min**	1 hr
6)	Repeat FE_{NO} and MEF FE_{NO}	30 min
7)	Spirometry (FEV_1)	10 min
	**Total Participant Time**	2 hr 50 min

4. Measure FE_{NO} and MEF FE_{NO} pre- and post-bronchodilator
5. Perform spirometry (FEV_1) pre- and post-bronchodilator

Inclusion Criteria

- Males or females aged 45 years and over
- Diagnosis of COPD with post-bronchodilator FEV_1 <80%
- Smoking history of greater than 10 pack-years

Exclusion Criteria

- Diagnosis of bronchiectasis or lung cancer
- Other co-morbidity likely to affect study participation
- Use of nasal steroid
- Inability to perform FE_{NO} testing

Assessments

Clinical assessment.

History of current and past respiratory symptoms, co-morbidities, medication, and smoking history will be taken. Blood pressure will be assessed once using a manual sphygmomanometer according to the standard technique [22].

FE_{NO} levels.

FE_{NO} measurements will be performed pre- and post-bronchodilator with a chemiluminescence analyser at an expiratory flow rate of 50 mL/s, as per the ATS guidelines [23].

MEF FE_{NO}.

Multiple expiratory flow FE_{NO} (MEF FE_{NO}) will be assessed pre- and post-bronchodilator using the technique described previously [24].

Spirometry.

Spirometry will be performed after measurements of exhaled nitric oxide before and after administration of 400mcg salbutamol via spacer, according to current standards [25].

Statistical analysis.

Pre- and post-bronchodilator FE_{NO} and FEV_1 measurements will be compared using paired t-tests, and the association between the change in FE_{NO} and FEV_1 will be evaluated by Spearman's rank correlation.

References

1. Broad J, Jackson R. Chronic obstructive pulmonary disease and lung cancer in New Zealand: a report prepared for the Thoracic Society of Australia and New Zealand. Report for the Thoracic Society of Australia and New Zealand. University of Auckland, 2003.

2. Vestbo J, Hurd SS, Agusti AG, Jones PW, Vogelmeier C, Anzueto A, et al. Global strategy for the diagnosis, management, and prevention of chronic obstructive pulmonary disease: GOLD executive summary. American journal of respiratory and critical care medicine. 2013;187(4):347-65. doi: 10.1164/rccm.201204-0596PP. PubMed PMID: 22878278.

3. Barnes P. Inhaled corticosteroids in COPD: a controversy. Respiration. 2010;80(2):89-95.

4. Magnussen H, Disse B, Rodriguez-Roisin R, Kirsten A, Watz H, Tetzlaff K, et al. Withdrawal of inhaled glucocorticoids and exacerbations of COPD. N Engl J Med. 2014;371(14):1285-94.

5. Suissa S, Ernst P, Vandemheen K, Aaron S. Methodological issues in therapeutic trials of COPD. Eur Respir J. 2008;31(5):927-33.

6. Yang I, Clarke M, Sim E, Fong K. Inhaled corticosteroids for stable chronic obstructive pulmonary disease. Cochrane Database Syst Rev. 2012;(7):CD002991.

7. Spencer S, Karner C, Cates C, Evans D. Inhaled corticosteroids versus long-acting beta2-agonists for chronic obstructive pulmonary disease. Cochrane Database Syst Rev. 2011;(12):CD007033.

8. Singh S, Amin A, Loke Y. Long-term use of inhaled corticosteroids and the risk of pneumonia in chronic obstructive pulmonary disease: a meta-analysis. Arch Intern Med. 2009;169(3):219-29.

9. Kew K, Seniukovich A. Inhaled steroids and risk of pneumonia for chronic obstructive pulmonary disease. Cochrane Database Syst Rev. 2014;(3):CD010115.

10. Deykin A, Halpern O, Massaro AF, Drazen JM, Israel E. Expired nitric oxide after bronchoprovocation and repeated spirometry in patients with asthma. American journal of respiratory and critical care medicine. 1998;157(3 Pt 1):769-75. doi: 10.1164/ajrccm.157.3.9707114. PubMed PMID: 9517589.

11. Weir D, Burge P. Effects of high dose inhaled beclomethasone dipropionate, 750 micrograms and 1500 micrograms twice daily, and 40 mg per day oral prednisolone on lung function, symptoms, and bronchial hyperresponsiveness in patients with non-asthmatic chronic airflow obstruction. Thorax. 1993;48(4):309-16.

12. Weir D, Gove R, Robertson A, Burge P. Corticosteroid trials in non-asthmatic chronic airflow obstruction: a comparison of oral prednisolone and inhaled beclomethasone dipropionate. Thorax. 1990;45(2):112-7.

13. Dummer J, Epton M, Cowan J, Cook J, Condliffe R, Landhuis C, et al. Predicting corticosteroid response in chronic obstructive pulmonary disease using exhaled nitric oxide. Am J Respir Crit Care Med. 2009;180(9):846-52.

14. Berry M, Shaw D, Green R, Brightling C, Wardlaw A, Pavord I. The use of exhaled nitric oxide concentration to identify eosinophilic airway inflammation: an observational study in adults with asthma. Clin Exp Allergy. 2005;35(9):1175-9.

15. Smith A, Cowan J, Brassett K, Filsell S, Mclachlan C, Monti-sheehan G, et al. Exhaled nitric oxide: a predictor of steroid response. Am J Respir Crit Care Med. 2005;172(4):453-9.

16. Haccuria A, Michils A, Michiels S, Van Muylem A. Exhaled nitric oxide: a biomarker integrating both lung function and airway inflammation changes. J Allergy Clin Immunol. 2014;134(3):554-9. PubMed Central PMCID: PMC2014.

17. Clini E, Bianchi L, Pagani M, Ambrosino N. Endogenous nitric oxide in patients with stable COPD: correlates with severity of disease. Thorax. 1998;53(10):881-3.

18. Papi A, Romagnoli M, Baraldo S, Braccioni F, Guzzinati I, Saetta M, et al. Partial reversibility of airflow limitation and increased exhaled NO and sputum eosinophilia in chronic obstructive pulmonary disease. Am J Respir Crit Care Med. 2000;162(5):1773-7.

19. Siva R, Green RH, Brightling CE, Shelley M, Hargadon B, McKenna S, et al. Eosinophilic airway inflammation and exacerbations of COPD: a randomised controlled trial. The European respiratory journal. 2007;29(5):906-13. doi: 10.1183/09031936.00146306. PubMed PMID: 17301099.

20. Doherty D, Belfer M, Brunton S, Fromer L, Morris C, Snader T. Chronic Obstructive Pulmonary Disease: consensus recommendations for early diagnosis and treatment. J Fam Pract. 2006;55(11):S1-S8.
21. Jones P, Harding G, Berry P, Wiklund I, Chen W, Kline Leidy N. Development and first validation of the COPD assessment test. Eur Respir J. 2009;34(3):648-54.
22. Beevers G, Lip G, O'Brien E. ABC of hypertension: blood pressure measurement: part II: conventional sphygmomanometry: technique of auscultatory blood pressure measurement. Br Med J. 2001;322(7293):1043-7.
23. Dweik R, Boggs P, Erzurum S, Irvin C, Leigh M, Lundberg J, et al. An official ATS clinical practice guideline: interpretation of exhaled nitric oxide levels (FEno) for clinical applications. Am J Respir Crit Care Med. 2011;184(5):602-15.
24. Condorelli P, Shin H, Aledia A, Silkoff P, George S. A simple technique to characterize proximal and peripheral nitric oxide exchange using constant flow exhalations and an axial diffusion model. J Appl Physiol. 2007;102(1):417-25.
25. Miller M, Hankinson J, Brusasco V, Burgos F, Casaburi R, Coates A, et al. Standardisation of spirometry. Eur Respir J. 2005;26(2):319-38.
