# Supplementary material for: Effect of Inhaled β2-Agonist on Exhaled Nitric Oxide in Chronic Obstructive Pulmonary Disease
Source: PLoS One. 2016 Jun 3;11(6):e0157019. doi: 10.1371/journal.pone.0157019 (PMC4892672; doi:10.1371/journal.pone.0157019)
Supplement: S1 Table — (PDF) [file pone.0157019.s002.pdf]

## Patient Demographics

| Study ID | Date | Age | Sex | Ethnicity | Height (cm) | Weight (kg) | Smoking hx | Pack-Years Smoking | CAT score | mMRC score | COPD exacerbations in past year | Hospitalisations for COPD in past year | Inhaled steroids | Total daily dose (BDP, mcg/day) |
|---|---|---|---|---|---|---|---|---|---|---|---|---|---|---|
| | | | Male=1 Female=2 | European=1 Maori=2 Other=3 | | | Current=1 Ex-smoker=2 | | | | | | Yes=1 No=0 | |
| 201 | 17-11-14 | 82 | 1 | 1 | 169 | 73.0 | 2 | 11 | 15 | 1 | 1 | 0 | 1 | 4000 |
| 203 | 24-11-14 | 69 | 2 | 1 | 168 | 70.7 | 2 | 19 | 19 | 1 | 1 | 0 | 1 | 800 |
| 204 | 25-11-14 | 66 | 1 | 1 | 173 | 71.7 | 2 | 30 | 22 | 0 | 0 | 0 | 1 | 250 |
| 205 | 26-11-14 | 73 | 1 | 1 | 171 | 72.4 | 2 | 23 | 14 | 2 | 0 | 0 | 0 | 0 |
| 206 | 27-11-14 | 54 | 2 | 1 | 159 | 75.4 | 1 | 10 | 11 | 2 | 0 | 0 | 1 | 1000 |
| 207 | 11-08-14 | 66 | 1 | 1 | 168 | 54.0 | 2 | 30 | 29 | 2 | 2 | 0 | 1 | 1000 |
| 208 | 03-09-14 | 72 | 1 | 1 | 170 | 88.5 | 2 | 40 | 29 | 3 | 2 | 0 | 1 | 1000 |
| 209 | 04-12-14 | 77 | 1 | 1 | 180 | 71.6 | 1 | 40 | 15 | 1 | 0 | 0 | 0 | 0 |
| 210 | 05-12-14 | 77 | 1 | 1 | 176 | 59.4 | 1 | 58 | 14 | 1 | 0 | 0 | 0 | 0 |
| 212 | 15-12-14 | 71 | 2 | 1 | 161 | 69.1 | 2 | 20 | 15 | 3 | 1 | 0 | 1 | 1000 |
| 213 | 18-12-14 | 57 | 2 | 1 | 168 | 84.7 | 2 | 30 | 23 | 3 | 3 | 2 | 1 | 800 |
| 215 | 07-01-15 | 73 | 1 | 1 | 180 | 98.6 | 2 | 50 | 26 | 3 | 3 | 3 | 1 | 1000 |
| 216 | 08-01-15 | 54 | 2 | 1 | 160 | 59.1 | 2 | 30 | 12 | 3 | 1 | 0 | 1 | 1000 |
| 218 | 13-01-15 | 79 | 1 | 1 | 150 | 47.5 | 1 | 59 | 13 | 0 | 3 | 0 | 1 | 2000 |
| 220 | 16-01-15 | 49 | 2 | 1 | 167 | 84.2 | 2 | 20 | 19 | 2 | 0 | 0 | 1 | 400 |
| 221 | 16-01-15 | 54 | 2 | 2 | 161 | 80.4 | 2 | 35 | 16 | 1 | 3 | 0 | 0 | 0 |
| 223 | 20-01-15 | 65 | 1 | 1 | 175 | 56.2 | 1 | 50 | 26 | 2 | 6 | 1 | 1 | 800 |
| 224 | 20-01-15 | 57 | 2 | 1 | 167 | 81.4 | 2 | 35 | 16 | 1 | 0 | 0 | 1 | 500 |
| 225 | 21-01-15 | 72 | 2 | 1 | 165 | 72.1 | 2 | 40 | 24 | 2 | 4 | 0 | 1 | 1000 |
| 226 | 21-01-15 | 75 | 1 | 1 | 180 | 86.1 | 2 | 32 | 24 | 3 | 2 | 0 | 1 | 500 |
| 227 | 23-01-15 | 77 | 2 | 1 | 160 | 50.1 | 2 | 21 | 20 | 1 | 1 | 1 | 1 | 1000 |

## PRE-BRONCHODILATOR FENO & SPIROMETRY

| Study ID | $FE_{NO}$ (ppb) | 50 mL/s | | MEF $FE_{NO}$ (ppb) | 100 mL/s | | 150 mL/s | | 200 mL/s | | 250 mL/s | | Spirometry | $FEV_1$ (L) | FVC (L) | % predicted $FEV_1$ |
|---|---|---|---|---|---|---|---|---|---|---|---|---|---|---|---|---|
| | | *Plateau 1* | *Plateau 2* | | *Plateau 1* | *Plateau 2* | *Plateau 1* | *Plateau 2* | *Plateau 1* | *Plateau 2* | *Plateau 1* | *Plateau 2* | | | | |
| 201 | | 22.4 | 24.6 | | 11.3 | 10.8 | 9.3 | 12.8 | 6.8 | 6.7 | 5.8 | 5.4 | | 1.10 | 2.46 | 48.7% |
| 203 | | 18.1 | 19.8 | | 8.7 | 8.6 | 6.8 | 6.4 | 5.0 | 5.7 | 5.2 | 4.9 | | 1.03 | 3.12 | 41.0% |
| 204 | | 40.2 | 38.2 | | 23.2 | 24.0 | 15.8 | 17.3 | 12.7 | 13.3 | 13.6 | 14.7 | | 1.44 | 3.71 | 45.6% |
| 205 | | 14.8 | 13.2 | | 5.3 | 7.0 | 6.2 | 5.0 | 4.6 | 4.5 | 4.4 | 4.4 | | 1.45 | 2.28 | 51.6% |
| 206 | | 9.8 | 9.8 | | 7.1 | 7.3 | 5.3 | 5.3 | 4.0 | 4.0 | 4.0 | 3.9 | | 1.10 | 3.50 | 42.6% |
| 207 | | 16.4 | 17.8 | | 9.8 | 9.4 | 7.6 | 7.8 | 5.4 | 5.4 | 5.8 | 5.1 | | 1.12 | 3.10 | 38.4% |
| 208 | | 85.5 | 87.7 | | 44.5 | 44.5 | 34.2 | 38.8 | 23.3 | 24.2 | 21.6 | 21.7 | | 1.11 | 1.84 | 39.6% |
| 209 | | 46.4 | 44.7 | | 21.4 | 18.8 | 17.2 | 16.4 | 13.5 | 13.5 | 12.9 | 12.4 | | 1.19 | 3.08 | 38.4% |
| 210 | | 10.0 | 9.8 | | 7.1 | 8.0 | 7.6 | 6.3 | 6.1 | 4.5 | 4.3 | 3.9 | | 1.04 | 2.73 | 35.9% |
| 212 | | 12.8 | 13.3 | | 6.3 | 6.0 | 5.8 | 5.2 | 6.3 | 5.5 | 4.4 | 4.4 | | 0.76 | 1.88 | 34.9% |
| 213 | | 11.5 | 11.5 | | 6.5 | 6.2 | 4.6 | 5.4 | 4.8 | 5.4 | 4.7 | 4.1 | | 0.55 | 1.50 | 19.4% |
| 215 | | 25.1 | 28.1 | | 14.7 | 12.0 | 10.3 | 10.0 | 7.0 | 8.3 | 6.7 | 7.3 | | 0.86 | 2.92 | 26.5% |
| 216 | | 15.0 | 15.8 | | 6.1 | 6.7 | 6.3 | 6.4 | 4.3 | 5.4 | 4.9 | 3.9 | | 0.64 | 2.17 | 24.4% |
| 218 | | 4.3 | 4.2 | | 3.9 | 3.8 | 3.5 | 3.8 | 3.4 | 3.3 | 3.7 | 3.0 | | 0.88 | 2.11 | 54.3% |
| 220 | | 16.0 | 15.0 | | 9.3 | 8.1 | 6.2 | 6.3 | 5.5 | 4.9 | 4.9 | 5.3 | | 0.73 | 1.56 | 24.3% |
| 221 | | 14.2 | 14.2 | | 7.9 | 7.5 | 6.4 | 5.7 | 4.7 | 5.4 | 4.7 | 4.5 | | 1.31 | 2.90 | 49.4% |
| 223 | | 8.6 | 8.8 | | 7.4 | 6.9 | 4.4 | 3.0 | 2.5 | 2.7 | 2.4 | 2.4 | | 1.01 | 2.29 | 30.6% |
| 224 | | 18.0 | 17.7 | | 9.4 | 10.5 | 7.7 | 8.4 | 7.4 | 6.5 | 6.2 | 5.9 | | 1.01 | 2.84 | 36.1% |
| 225 | | 12.9 | 12.7 | | 7.7 | 6.6 | 5.9 | 5.4 | 4.6 | 4.4 | 5.3 | 5.0 | | 0.61 | 1.74 | 26.5% |
| 226 | | 45.3 | 53.3 | | 25.3 | 24.0 | 19.9 | 18.8 | 16.5 | 14.8 | 14.5 | 14.4 | | 0.82 | 2.88 | 25.8% |
| 227 | | 8.5 | 8.2 | | 5.9 | 6.6 | 5.4 | 5.1 | 4.3 | 4.1 | 3.9 | 3.9 | | 1.25 | 1.97 | 64.1% |

## POST-BRONCHODILATOR FENO & SPIROMETRY

| Study ID | $FE_{NO}$ (ppb) | 50 mL/s | | MEF $FE_{NO}$ (ppb) | 100 mL/s | | 150 mL/s | | 200 mL/s | | 250 mL/s | | Spirometry | $FEV_1$ (L) | FVC (L) | % predicted $FEV_1$ |
|---|---|---|---|---|---|---|---|---|---|---|---|---|---|---|---|---|
| | | *Plateau 1* | *Plateau 2* | | *Plateau 1* | *Plateau 2* | *Plateau 1* | *Plateau 2* | *Plateau 1* | *Plateau 2* | *Plateau 1* | *Plateau 2* | | | | |
| 201 | | 20.1 | 19.8 | | 8.3 | 8.8 | 8.7 | 8.2 | 5.6 | 6.9 | 5.1 | 6.0 | | 1.23 | 2.43 | 54.4% |
| 203 | | 18.2 | 17.3 | | 10.1 | 8.8 | 6.0 | 5.6 | 4.9 | 4.6 | 4.7 | 4.4 | | 1.10 | 3.34 | 43.8% |
| 204 | | 41.4 | 37.9 | | 23.0 | 22.0 | 14.6 | 14.1 | 13.9 | 12.5 | 13.6 | 11.8 | | 1.74 | 4.12 | 55.1% |
| 205 | | 16.1 | 14.8 | | 7.7 | 7.7 | 5.0 | 4.6 | 5.0 | 5.8 | 4.1 | 4.6 | | 1.60 | 2.94 | 56.9% |
| 206 | | 13.5 | 14.2 | | 8.7 | 7.0 | 5.0 | 4.7 | 4.0 | 4.0 | 3.6 | 3.5 | | 1.15 | 3.48 | 44.6% |
| 207 | | 17.3 | 15.3 | | 9.0 | 8.1 | 6.6 | 6.5 | 5.4 | 5.8 | 5.1 | 5.0 | | 1.12 | 3.12 | 38.4% |
| 208 | | 84.5 | 87.1 | | 40.8 | 38.0 | 32.1 | 31.6 | 30.7 | 28.2 | 21.0 | 21.1 | | 1.59 | 2.46 | 56.8% |
| 209 | | 54.5 | 56.7 | | 26.9 | 25.7 | 18.2 | 17.5 | 14.6 | 13.9 | 12.4 | 12.7 | | 1.53 | 4.11 | 49.4% |
| 210 | | 14.1 | 13.3 | | 11.4 | 10.3 | 5.5 | 7.4 | 7.0 | 5.7 | 5.2 | 5.8 | | 1.21 | 3.22 | 41.7% |
| 212 | | 14.5 | 15.3 | | 7.6 | 7.4 | 5.6 | 5.1 | 4.7 | 5.5 | 3.6 | 4.5 | | 0.84 | 2.36 | 38.5% |
| 213 | | 17.9 | 16.0 | | 9.2 | 8.2 | 6.1 | 6.4 | 5.9 | 5.5 | 5.5 | 5.0 | | 0.65 | 2.11 | 22.9% |
| 215 | | 26.8 | 29.1 | | 15.4 | 14.1 | 8.6 | 7.6 | 8.3 | 6.5 | 5.1 | 5.6 | | 1.40 | 3.62 | 43.1% |
| 216 | | 19.9 | 20.0 | | 8.0 | 7.3 | 6.9 | 7.2 | 5.1 | 5.3 | 5.1 | 4.4 | | 0.79 | 2.20 | 30.2% |
| 218 | | 5.0 | 4.5 | | 3.1 | 3.2 | 3.3 | 3.4 | 2.7 | 3.0 | 2.7 | 3.1 | | 0.89 | 1.94 | 54.9% |
| 220 | | 14.7 | 14.1 | | 8.6 | 7.6 | 6.2 | 5.5 | 5.4 | 5.5 | 5.0 | 4.5 | | 0.78 | 1.73 | 26.0% |
| 221 | | 14.8 | 14.5 | | 7.4 | 7.0 | 5.6 | 5.1 | 4.7 | 4.5 | 4.3 | 4.0 | | 1.53 | 3.46 | 57.7% |
| 223 | | 13.6 | 12.3 | | 7.4 | 4.5 | 3.9 | 2.6 | 2.3 | 2.5 | 2.1 | 2.9 | | 1.00 | 2.30 | 30.3% |
| 224 | | 22.8 | 23.3 | | 10.5 | 10.9 | 8.2 | 8.4 | 6.8 | 7.0 | 5.8 | 5.1 | | 1.18 | 3.49 | 42.1% |
| 225 | | 18.3 | 18.1 | | 9.7 | 9.9 | 9.3 | 7.2 | 5.7 | 6.4 | 6.3 | 6.2 | | 0.67 | 1.91 | 29.1% |
| 226 | | 54.0 | 53.0 | | 26.1 | 27.7 | 20.5 | 19.7 | 14.8 | 12.8 | 14.0 | 14.5 | | 0.83 | 2.94 | 26.1% |
| 227 | | 7.6 | 8.3 | | 5.5 | 5.4 | 4.5 | 4.2 | 3.8 | 3.3 | 4.0 | 3.2 | | 1.29 | 1.76 | 66.2% |

## AUC-NO*

*over 10 sec exhalation period

| | Pre-bronchodilator | | | | Post-bronchodilator | | | |
|---|---|---|---|---|---|---|---|---|
| | 50 mL/s | | 200 mL/s | | 50 mL/s | | 200 mL/s | |
| **Study ID** | *Exhalation 1* | *Exhalation 2* | *Exhalation 1* | *Exhalation 2* | *Exhalation 1* | *Exhalation 2* | *Exhalation 1* | *Exhalation 2* |
| 201 | 248.7 | 255.0 | 65.3 | 59.5 | 212.4 | 205.3 | 57.5 | 68.9 |
| 203 | 168.4 | 198.8 | 68.4 | 54.8 | 196.2 | 158.5 | 55.8 | 49.2 |
| 204 | 386.7 | 420.7 | 166.3 | 147.2 | 347.6 | 450.4 | 142.5 | 115.0 |
| 205 | 155.8 | 164.8 | 52.4 | 66.2 | 164.3 | 145.4 | 57.8 | 55.1 |
| 206 | 120.2 | 93.3 | 41.1 | 41.6 | 114.2 | 122.0 | 36.1 | 39.6 |
| 207 | 150.5 | 161.6 | 50.8 | 50.8 | 142.5 | 135.4 | 46.5 | 50.7 |
| 208 | 458.5 | 456.3 | 272.9 | 246.5 | 753.1 | 836.7 | 323.4 | 265.5 |
| 209 | 511.1 | 417.0 | 159.6 | 146.0 | 526.4 | 518.9 | 184.4 | 150.1 |
| 210 | 106.3 | 120.8 | 59.7 | 37.6 | 130.2 | 134.9 | 65.2 | 51.9 |
| 212 | 126.2 | 136.9 | 63.9 | 53.7 | 157.8 | 147.1 | 45.3 | 53.0 |
| 213 | 134.7 | 126.2 | 44.5 | 47.7 | 263.1 | 161.4 | 60.4 | 51.1 |
| 215 | 261.4 | 233.5 | 70.6 | 87.5 | 316.6 | 205.5 | 97.5 | 73.6 |
| 216 | 149.6 | 165.1 | 54.2 | 51.9 | 183.3 | 183.0 | 54.9 | 52.8 |
| 218 | 58.8 | 83.0 | 36.9 | 33.0 | 51.3 | 40.4 | 27.0 | 31.9 |
| 220 | 161.6 | 149.8 | 50.8 | 49.6 | 142.7 | 142.2 | 51.0 | 51.9 |
| 221 | 147.5 | 145.9 | 55.7 | 52.9 | 142.0 | 144.1 | 51.2 | 47.9 |
| 223 | 62.1 | 53.1 | 22.3 | 27.4 | 120.7 | 107.1 | 17.8 | 23.9 |
| 224 | 215.1 | 184.6 | 73.3 | 64.9 | 222.0 | 226.9 | 67.4 | 69.9 |
| 225 | 129.2 | 128.1 | 42.5 | 44.8 | 140.2 | 183.2 | 53.1 | 73.0 |
| 226 | 496.8 | 530.5 | 158.9 | 148.6 | 495.9 | 503.5 | 150.3 | 138.4 |
| 227 | 84.6 | 123.6 | 39.2 | 42.3 | 108.7 | 85.3 | 36.9 | 33.0 |
